# Quality of life in children with sleep-disordered breathing

**DOI:** 10.5935/1808-8694.20120003

**Published:** 2015-11-20

**Authors:** Amaury de Machado Gomes, Otávio Marambaia dos Santos, Kleber Pimentel, Pablo Pinillos Marambaia, Leonardo Marques Gomes, Márcia Pradella-Hallinan, Manuela Garcia Lima

**Affiliations:** a Specialist in ENT and Traffic Medicine (MSc. in Medicine and Human Health at the Bahia Medical School, Salvador - BA. Medical Advisor in the Inooa/ABORL-CCF ENT Specialization Internship Program); b PhD Student at Porto medical School - Portugal (Professor at the Bahia School of Medicine and Public Health (EBMSP), Salvador - BA, Coordinator in the Inooa/ABORL-CCF ENT Specialization Internship Program); c MSc. in Internal Medicine (UFBA). (Medical Advisor in the Inooa/ABORL-CCF ENT Specialization Internship Program. Professor at the Bahia School of Medicine and Public Health (EBMSP), Salvador - BA); d Specialist in ENT (MSc. in Medicine and Human Health at the Bahia School of Medicine and Public Health (EBMSP), Salvador - BA. Medical Advisor in the Inooa/ABORL-CCF ENT Specialization Internship Program); e MD graduated at the Bahia School of Medicine and Public Health (EBMSP), Salvador - BA. (MD); f PhD in Sciences at the Federal University of Sao Paulo (UNIFESP) (MD in the Pediatric Polysomnography Course of the Department of Psychobiology at UNIFESP-EPM); g PhD in Public Health at the Federal University of Bahia (UFBA) (Professor at the Bahia School of Medicine and Public Health (EBMSP), Salvador - BA, Brazil)

**Keywords:** child, quality of life, sleep apnea, obstructive, sleep disorders, snoring

## Abstract

Children may present sleep-disordered breathing (SDB) and suffer with adverse effects upon their quality of life.

**Objective:**

This study assessed the quality of life of children with SDB, compared subjects with obstructive sleep apnea syndrome (OSAS) and primary snoring (PS), and identified which areas in the OSA-18 questionnaire are more affected.

**Methods:**

This is a historical cohort cross-sectional study carried out on a consecutive sample of children with history of snoring and adenotonsillar hyperplasia. The subject's quality of life was assessed based on the answers their caregivers gave in the OSA-18 questionnaire and on diagnostic polysomnography tests.

**Results:**

A number of 59 children participated in this study with mean age of 6.7 ± 2.26 years. The mean score of the OSA-18 was 77.9 ± 13.22 and the area most affected were “caregiver concerns” (21.8 ± 4.25), “sleep disturbance” (18.8 ± 5.19), “physical suffering” (17.3 ± 5.0). The impact was low in 6 children (10.2%), moderate in 33 (55.9%) and high in 20 (33.9%). PS was found in 44 children (74.6%), OSAS in 15 (25.6%). OSAS had higher score on “physical suffering” area than PS (*p* = 0.04). The AI (r = 0.22; *p* = 0.08) and AHI (r = 0.14; *p* = 0.26) were not correlated with OSA-18.

**Conclusion:**

Sleep disordered breathing in childhood cause impairment in quality of life and areas most affected the OSA-18 were: “caregiver concerns”, “sleep disturbance” and “physical suffering”. OSAS has the domain “physical suffering” more affected than primary snorers.

## INTRODUCTION

Sleep-disordered breathing (SDB) is relatively frequent in the pediatric population and includes primary snoring (PS) and obstructive sleep apnea syndrome (OSAS).

PS is defined as respiratory noise, but the architecture of sleep, alveolar ventilation, and blood oxygen levels are normal. It is found in 7% to 9% of the children aged between one and 10 years^1^.

Obstructive sleep apnea syndrome (OSAS) in children is a sleep respiratory disorder characterized by prolonged partial obstruction of the upper airways or intermittent complete obstruction (obstructive apnea) episodes that hamper normal ventilation during sleep and adversely affect normal sleep patterns. Epidemiological studies have observed pediatric prevalence rates ranging between 0.7% and 3%. Incidence rates peak among preschoolers aged between two and six years. Genders are affected equally, and adenotonsillar hypertrophy is the main cause of OSAS^2^.

Children with OSAS may also have obstructive hypoventilation, a condition characterized by episodes of snoring and paradoxical motion of the rib cage lasting for various minutes without apnea^3^.

OSAS may have severe consequences such as cor pulmonale, delays in weight and height gain, behavior and learning disorders, and other cognitive impairments^4^. Adenotonsillectomy cures 75% to 100% of the children with adenotonsillar hypertrophy and is the first line of treatment for OSAS^5^.

Clinical history and physical examination stand as the most widely used methods to diagnose children with SDB. When combined, they are valid only for screening patients that require additional investigation. These tools are characterized by low sensitivity and specificity and cannot be used to predict OSAS^6^^,^^7^.

Polysomnography (PSG) testing done in a sleep lab is considered the best diagnostic method (gold standard) and can be performed in patients of all ages. It is specially recommended to differentiate OSAS from PS, central apnea, nocturnal seizure, and narcolepsy, aside from being useful in assessing OSAS severity, risk of immediate postoperative complication, and post-treatment follow-up^7^^,^^8^.

Pediatric SDB leads to significant clinical repercussions and may affect patient quality of life (QOL), an indicator that can be measured by interviewing individuals with the aid of proper questionnaires^9^, ^10^, ^11^.

The impact upon the quality of life of children with SDB and enlarged pharyngeal tonsils has been a topic of interest in recent studies. International studies have shown that SDB poses significant adverse impacts upon the QOL of children with adenotonsillar hypertrophy^12^, ^13^, ^14^, ^15^, ^16^, ^17^, ^18^, ^19^ and that they improve after surgery.

The few papers written in Brazil about this subject have shown that the QOL of children with SDB cis compromised^20^, ^21^, ^22^, ^23^, ^24^, ^25^. However, they fail to establish differential diagnosis, disease severity, and the possible differences between levels of involvement. Silva et al.^20^ published the first study on quality of life o children with SDB in Brazil using questionnaire OSA-18, based on the paper by Franco et al.^26^.

This study aims to analyze a population of children with sleep-disordered breathing and assess their quality of life using the OSA-18 questionnaire and polysomnography testing.

## METHOD

This is historical cohort cross-sectional study enrolled underprivileged children aged between three and 12 years. The sample was selected consecutively at the mouth breathing ward of an ENT care reference center between August of 2008 and March of 2009. This study was approved by the institution's Ethics Committee and given permit n° 34/2008.

Children of both genders aged between three and 12 years of age were enrolled in this study. The subjects had history of snoring or apnea lasting over four months, and palatine or pharyngeal tonsil hyperplasia on physical examination, and underwent polysomnography testing. Their parents or guardians were required to sign a free informed consent term. Children with craniofacial malformation, psychiatric or behavioral disorders, altered psychomotor development, taking medication that acts upon the central nervous system, immunocompromised subjects, and with previous adenotonsillectomy were excluded.

Patient and primary caregiver (parent or guardian) data were collected in a form containing clinical and sociodemographic information. Age was measured in years based on patient birth dates. Ethnicity was defined based on census data, being skin color used as a reference. Other variables such as duration of sleep disorder in years, reported symptoms, if the child sleeps in separate room, and degree of education of the primary caregiver were also considered.

Caregivers were asked to answer another standard questionnaire on sleep disorders prior to polysomnography testing.

The physical examination consisted of examination of the oral cavity, anterior rhinoscopy, otoscopic examination, and laryngoscopy. Examination was performed by the main author. The positions of palate and base of the tongue were defined through oral cavity examination based on the modified classification described by Mallampati^27^. Subjects were rated from classes I to IV based on the degree the soft palate could be visualized in relation to the base of the tongue. The size of the palatine tonsils was measured based on the categorization proposed by Brodsky^28^ and degree of obstruction was rated into grades I (palatine tonsils occupy up to 25% of the oropharyngeal space), II (palatine tonsils occupy between 26% and 50% of the oropharyngeal space), III (palatine tonsils occupy between 51% and 75% of the oropharyngeal space), and IV (palatine tonsils occupy more than 75% of the oropharyngeal space). Patients on grades III and IV were considered to have obstructive tonsils^29^.

Endoscopic examination was used to rate the degree of obstruction by adenoids into grades I (up to 25%), II (26% to 50%), III (51% to 75%), and IV (> 75%). Subjects were accompanied by their caregivers and were not given medication during examination. A flexible 3.2 mm diameter Machidda ENT P III endoscope connected to a 250 W Endoview light source, a Toshiba CCD IKCU 44A camera, a Sony KU 1441B monitor, and a Sony SLV 40BR VCR were used.

A precision mechanical scales was used to measure the subjects' weight and height.

The subjects' quality of life was assessed based on the answers given by the children's caregivers to questionnaire OSA-18 adapted to Brazilian Portuguese by Silva et al.^20^ through the technique of back-translation, to attain exact compliance with the terms used in the original document.

This questionnaire consists of 18 items grouped into five domains. Each item is given a score ranging from one to seven (Chart 1).

**Chart 1:** Model of the OSA-18 Quality of Life Assessment Questionnaire.

	Never	Almost never	A few times	Sometimes	Many times	Most of the times	All the time
1. Sleep disorder							
Loud snoring?	1	2	3	4	5	6	7
Periods at night when you held your breath or stopped breathing?	1	2	3	4	5	6	7
Gagging noise or panting while sleeping?	1	2	3	4	5	6	7
Restless sleep or frequent awakenings during sleep?	1	2	3	4	5	6	7
2. Physical distress							
Nasal breathing because of nasal obstruction?	1	2	3	4	5	6	7
Frequent colds or upper airway infections?	1	2	3	4	5	6	7
Nasal secretion or a running nose?	1	2	3	4	5	6	7
Difficulty feeding?	1	2	3	4	5	6	7
3. Emotional distress							
Mood change or anger fit?	1	2	3	4	5	6	7
Aggressive or hyperactive behavior?	1	2	3	4	5	6	7
Discipline problems	1	2	3	4	5	6	7
4. Daily problems							
Excessive daytime sleepiness or frequent naps?	1	2	3	4	5	6	7
Little concentration or attention?	1	2	3	4	5	6	7
Difficulty to wake up in the morning?	1	2	3	4	5	6	7
5. Concern of the caretakers							
Make you worry about your child's general health?	1	2	3	4	5	6	7
Create the concern that your child is not breathing enough air?	1	2	3	4	5	6	7
Impact on your capacity to execute your daily activities?	1	2	3	4	5	6	7
Make you feel frustrated?	1	2	3	4	5	6	7
Total score OSA(18-126)							

Total OSA-18 scores may range from 18 to 126 and are categorized into three groups depending on the level of impact upon quality of life, as follows: small: (under 60), moderate (60 to 80); and major (above 80). The more frequently each of the domain items occur, the higher is the final score and so the adverse repercussions upon quality of life. This instrument also contemplates a global rate connected to quality of life in a scale from zero to ten assigned by the caregivers, in which zero means lowest quality of life and ten the highest quality of life possible. Comparisons were made between the total scores in the five domains of the OSA-18 and the global ratings for quality of life for variables gender, age, complaint duration, OSAS, and PS. The questionnaires included in this study were submitted to the same caregiver (parent or guardian) by the min author.

The diagnosis for PS or OSAS resulted from full-night polysomnography tests carried out at a sleep lab. The subjects were placed in a silent room with air-conditioning (split type) to sleep spontaneously without sedation accompanied by their caregivers. Caregivers were asked to answer a questionnaire on their children's sleep disorders before the tests were performed. Subjects were advised to avoid stimulants (coffee, chocolate, soft drinks) on the day of the test. Tests were done using a 16-channel Alice 3 Healthdyne Respironics containing electroencephalography (C3/ A2), (C4/A2), (O1/A2), O2/A1), submental and tibial electromyography, and right and left electrooculography systems. Electrodes were placed based on the 10-20 international system, and patients were equipped with a nasal thermistor Airflow Sensor 6210, belts to record abdominal and chest effort, a microphone fitted to the neck area to acquire snore sounds, a Heathdyne Technologies pulse oximeter to measure oxygen saturation, electrocardiography devices to measure heart rate, and a bed position sensor. The thermistors were placed in front of the mouth and nose to record nasal and oral airflow, as the studied subjects were predominantly mouth breathers.

Sleep architecture was assessed through the standard technique and the amount of time spent on each sleep stage was expressed as a percentage of the total time of sleep^2^.

Central apnea was defined as the absence of oral and nasal airflow measured by a nasal thermistor in the absence of respiratory effort. Episodes lasting for 10 seconds or more were considered. Obstructive apnea was defined as the absence of oral and nasal airflow measured by a nasal thermistor with present chest and abdominal movements for at least two respiratory cycles. Mixed apnea was defined as cases in which central and obstructive components were observed in any given order, with central component episodes lasting for three seconds. Hypopnea was defined as a reduction of 50% in oral and nasal airflow amplitude measured by a thermistor in combination with oxyhemoglobin desaturation > 3%, or SpO_2_ < 90%, and/or awakenings^4^. The identification and classification of sleep stages were based on 30-second epochs according to the criteria described by Rechtschaffen & Kales^30^.

The following PSG respiratory variables were analyzed to categorize the events:

Apnea index (AI): number of obstructive and mixed apnea episodes lasting for at least two respiratory cycles expressed in events per hour; apnea-hypopnea index (AHI): summation of the number of obstructive and mixed apnea episodes expressed in events per hour; and oxygen saturation nadir (SpO_2_ nadir): minimum oxygen saturation during sleep test measured on pulse oximeter. Respiratory effort related arousal (RERA) was not analyzed in this study.

All PSG tests were analyzed by an experienced physician in accordance with the classification of events and pediatric criteria dictated by the American Thoracic Society^2^.

The subjects were divided into primary snorers (without apnea), when they had AI < 1 event/hour, and those with OSAS (with apnea), when the AI was > 1 event/hour. The children with OSAS were further divided into mild (1 < AI < 5), moderate (5 < AI < 10) and severe (IA > 10) OSAS. The data obtained were correlated with sociodemographic variables and OSA-18 </IA scores and domains.

Quality of life was assessed using the OSA-18 questionnaire, while SDB was rated based on AI, AHI, and SpO_2_ nadir.

Software program Statistical Package for the Social Sciences (SPSS Chicago-IL, version 11.0) was used to build the database and statistical analysis. Epi Info version 6 and PEPI “(*computer program for epidemiologists*)” Version 4.04x were used to calculate the sample.

Continuous variables were presented in the form of mean values and standard deviations and median values. Categorical variables were expressed as ratios (relative frequency). Independent samples were compared through Student's t-test or the Mann-Whitney test when data was not normally distributed. Comparisons were made between overall scores and scores in each of the domains attained by each group. Pearson's chi-squared test was used in categorical and association variables and Fisher's exact test was used for variables that failed to meet the criteria required by Pearson's test. Tests were two-tailed and adopted a level of statistical significance of 5%.

## RESULTS

One hundred and ten children were examined between August of 2008 and March of 2009. Sixty-three agreed to join the study and four failed to undergo PSG. The study enrolled 59 children, 55.9% of whom (n = 33) were females. Mean age at the time of enrollment was 6.77 ± 2.26 years. Ten caregivers (16.9%) informed their children were Caucasian, 43 (72.9%) brown, and six (10.2%) black; 49 (83.1%) were non-Caucasians.

According to the caregivers, the mean duration of sleep disorders in the children enrolled in the study was 3.94 ± 1.77 years. All 59 (100%) subjects included the study were snorers and the most frequently reported symptoms were mouth breathing (100%), nasal obstruction (93.8%), and agitated sleep (91.7%).

ENT examination revealed a predominance of grades III and IV for items palatine and pharyngeal tonsil obstruction, as follows:

1 Palatine tonsils
  • Grade II: 14 (23.7%);
  • Grade III: 28 (47.5%);
  • Grade IV: 17 (28.8%).
2 Pharyngeal tonsils
  • Grade I: 1 (1.7%);
  • Grade II: 16 (27.1%);
  • Grade III: 29 (49.2%);
  • Grade IV: 13 (22%).

Physical examination showed that 54 (91.5%) subjects were given Class I Mallampati scores. Anterior rhinoscopy revealed enlarged turbinates in 21 children (35.6%) and five (8.5%) subjects with deviated septa. Ear examination findings were normal in 43 (72.9%) children, 12 (20.4%) had wax plugs, and four (6.7%) had cloudy tympanic membranes.

In their households, 32 (54.2%) children shared a room with their caregivers and 54 (91.5%) slept for nine to 11 hours a day. Twenty-seven (45.8%) were attending a day care center or pre-school, nine (15.3%) were on the first grade, eight (13.6%) on second grade, nine (15.3%) on third grade, three (5.13%) on fourth grade, one (1.7%) on fifth grade, and two (3.4%) did not go to school.

Social and demographic information and the answers to the OSA-18 questionnaire were provided by the children's primary caregivers. Fifty-three (89.8%) of the caregivers were the children's mothers, two (3.4%) were their fathers, and four (6.8%) were others. The caregiver's mean age was 32.5 ± 6.20 years.

Caregiver level of schooling was reported as follows: 33 (55.9%) completed second grade school (dropped out of college + completed second grade), 22 (37.3%) completed first grade school (completed first grade + dropped out of second grade), and four (6.8%) failed to complete first grade school (dropped out of first grade + illiterate).

Forty-two (71.2%) of the caregivers reported they were married, 13 (22%) were single, and four (6.8%) had other marital statuses.

Fifty-two (88.1%) reported their household income amounted to up to two minimum wages. Mean household income was 1.1 ± 0.32 minimum wage. The mean number of persons in the household supported by such income was 3.8 ± 1.00 persons.

**Graph 1:**
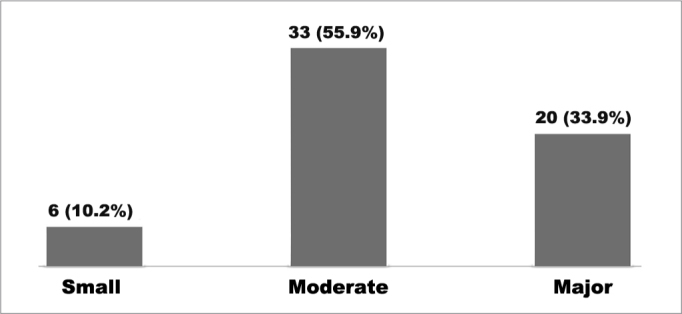
Impact on the quality of life in children seen between August of 2008 and March of 2009; n (%).

All 59 caregivers completed the OSA-18 questionnaire without trouble. The mean score at the time of enrollment was 77.9 ± 13.22. The impact on quality of life brought by the disorder was rated as small in 6 (10.2%) cases, moderate in 33 (55.9%), and major in 20 (33.9%) (Graph 1).

The OSA-18 domain with the highest mean score was “concern of persons in charge” (21.8 ± 4.25), followed by “sleep disturbance” (18.8 ± 5.19), “physical suffering” (17.3 ± 5.0), “emotional suffering” (11.8 ± 4.52), and “diurnal problems” (8.0 ± 4.0). The mean global score for quality of life was 5.35 ± 1.45.

Forty-four (74.6%) children were primary snorers, while 15 (25.4%) had OSAS (apnea). Full-night PSG indicated that six (10.2%) children had mild OSAS, one (1.7%) had moderate OSAS, and eight (13.6%) had severe OSAS.

No statistically significant differences were found between the OSAS and PS groups in regards to caregiver social and demographic traits (Table 1, Table 2).

**Table 1:** Socio-demographic and anthropometric characteristics of the population with primary snoring and sleep apnea studied between August of 2008 and March of 2009; n (%).

Variable name	Apnea n = 15	Primary snoring n = 44	*p*-value
Gender			
Males	06 (40%)	20 (45.5%)	
Females	09 (60%)	24 (54.5%)	
Informer (caretaker)			
Mother	13 (86.6%)	40 (90.9%)	
Father	0.1 (6.7%)	0.1 (2.3%)	1.0
Others	0.1 (6.7%)	0.3 (6.8%)	
Caretaker's marital status			
Single	0.5 (33.4%)	08 (18.2%)	
Married	08 (53.3%)	34 (77.3%)	0.18
Others	02 (13.3%)	02 (4.5%)	
Caretaker's educational level			
Fundamental incomplete	01 (6.6%)	03 (6.8%)	
Fundamental complete	03 (20%)	19 (43.2%)	0.25
High School complete	11 (73.4%)	22 (50%)	
Family income			
< 2 minimum wages	13 (86.6%)	39 (88.6%)	
≥ 2 minimum wages	02 (13.4%)	05 (11.4%)

**Table 2:** Socio-demographic characteristics of the population assessed between August of 2008 and March of 2009; (Mean ± SD).

Variable name	Apnea n = 15	Primary snoring n = 44	*p*value
Children's ages (years)			
Mean ± SD	5.9 ± 2.25	7.0 ± 2.21	0.08
Duration of complaints (years)			
Mean ± SD	3.7 ± 2.25	4.0 ± 1.70	0.59
Caretaker's age (years)			
Mean ± SD	32. ± 4.99	32.6 ± 6.61	0.85
Caretaker's education (in years)			
Mean ± SD	11.8 ± 2.50	10.6 ± 3.51	0.20

Subjects with apnea (OSAS) had higher mean total scores on OSA-18 than primary snorers, but this finding was not statistically significant (*p* = 0.08). Children with OSAS were more affected by the “physical suffering” domain on OSA-18 than primary snorers (*p* = 0.04). Both groups had similar most frequently mentioned domains (Table 3).

**Table 3:** Frequency of the domain mean scores and the total OSA-18 score of children with apnea and snoring seen between August of 2008 and March of 2009; (Mean ± SD).

Domains and scores	Apnea n = 15	Snoring n = 44	*p*value
Sleep disorder	20.2 ± 5.68	18.3 ± 5	0.20
Physical distress	19.6 ± 5.56	16.6 ± 4.70	0.04
Emotional distress	11.2 ± 5.18	12 ± 4,32	0.59
Daytime problems	9.1 ±4.76	7.6 ±3.77	0.22
Caretakers' concerns	22.9 ± 4.25	21.4 ± 4.23	0.23
OSA-18 total score	83 ± 15.08	76.2 ± 2.24	0.08
Global score	2.46 ± 0.43	2.15 ± 0.60	0.10

**Graph 2:**
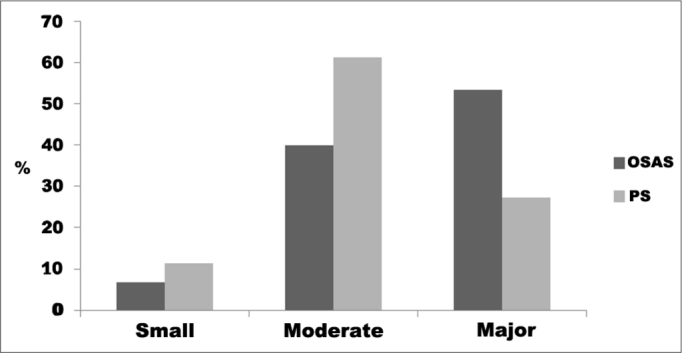
Frequency of the degree of impact on quality of life according to OSA-18 in children with OSAS and PS seen between August of 2008 and March of 2009 (*p* = 0.18).

The degree of impact on quality of life represented by the mean OSA-18 scores for groups OSAS and PS were: small in one (6.7%) and five (11.3%) cases; moderate in six (40%) and 27 (61.3%) cases; and major in eight (53.3%) and 12 (27.2%) respectively. No statistically significant difference was found between groups (*p* = 0.18), as seen on Graph 2.

## DISCUSSION

Quality of life is increasingly recognized as an important measure of care outcome in clinical medicine. However, the impact of OSAS on the QOL of children has been underestimated^18^. This study aimed to evaluate this issue based on a sample of 59 children with signs and symptoms of SDB aged between three and 12 years (mean age 6.77 ± 2.26 years). Fifteen (25.4%) children had a confirmed diagnosis of OSAS after undergoing PSG.

A national epidemiological study verified high prevalence rates of sleep-disordered breathing in 998 underprivileged students aged between nine and 14 years. Snoring was observed in 27.6% of the subjects, a rate significantly higher than what has been observed in other countries among children of similar age ranges^31^.

It is estimated that the prevalence rate of PS in the pediatric population ranges between 3.2% and 12.1%. OSAS affects fewer children, with prevalence rates ranging between 0.7% and 10.3%. As many as 70%^32^ of the children with SDB sent for PSG examination are diagnosed with OSAS. The literature presents a wide range of OSAS prevalence rates in children with a clinical history of SDB^29^^,^^33^, ^34^, ^35^, ^36^.

This study included primary snorers (100%) frequently affected by the classic symptoms of SDB, mouth breathers (100%), subjects with nasal obstruction (93.2%), agitated sleep (91.5%), upper respiratory tract infection (83.1%), hyperactivity disorder (81.4%), sweating (78%), sialorrhea (76,3%), and reported apnea (69.5%). The least frequently seen symptoms were physical development (23.7%), followed by diurnal sleepiness (11.9%). Ramos et al.^37^ found similar results in a Brazilian sample, with predominance of snoring (93.5%), nasal obstruction (93.5%), and agitated sleep (88.2%). The presence of symptoms on children with OSAS and PS was similar. Mouth breathing and nasal symptoms were the most commonly found by other authors^20^^,^^26^.

SDB leads to impacts on the quality of life of affected children and their caregivers, as seen in may studies published internationally. Franco et al.^26^ showed moderate and major impact on the QOL of 67% of the children in their sample. Michell & Kelly^38^ found moderate and major impact on the QOL of 72% of the children with OSAS. Goldstein et al.^36^ studied 64 children and found 66% of moderate and major impact on QOL. In Brazil, Silva et al.^20^ found moderate and major impact in 97.9% of the subjects. In our study the degree of impact on quality of life verified in the OSA-18 survey was small in six (10.2%), moderate in 33 (55.9%) and major in 20 (33.9%) cases, i.e., 53 (89.8%) children enrolled in the study had moderate to major levels of impact on QOL. This finding is consistent with other studies in the literature.

No statistically significant differences were found when the OSAS (apnea) group was compared to the PS (no apnea) group in terms of impact on quality of life (*p* = 0.18), as also reported by Mitchell & Kelly^38^.

Over half (54.2%) of the children slept in the same room as their caregivers. The children's mothers answered the questionnaires in 89.8% of the cases; 50.8% of the caregivers completed second grade and went to school for 10.9 ± 3.31 years. These findings were similar to what Silva et al.^20^ reported. In their study, 62.5% of the children shared a room with their caregivers, over 80% of the questionnaires were responded by the children's mothers, and caregivers stayed in school for 8.2 years (SD = 3.14).

Mean time of complaints in this study was 3.9 years, against the 4.6 reported by Silva et al.^20^.

It is possible that these results are a translation of the difficulty these children have finding ENT care in northeastern Brazil, when compared to the two years reported by Franco et al.^26^, a fact that possibly portrays the reality in North America.

In this study, higher mean scores were observed in OSA-18 domains “concern of persons in charge”, “sleep disturbance”, and “physical suffering”. The items that make up these domains involve issues related to the most common aspects of SDB (snoring, choking, agitated sleep, mouth breathing, upper airway infection, difficulty feeding, concern of the parents with their child's health). These results are similar to what Silva et al.^20^ reported. Other authors listed the same domains (“sleep disturbance”, followed by “concern of persons in charge” and “physical suffering”) as the most affected^26^^,^^39^^,^^40^. The least affected domain on OSA-18 observed in this study was “diurnal problems”, as also seen in other Brazilian studies by Silva et al.^20^ and Nascimento et al.^21^, and differently from what was seen in international studies, in which domain “emotional problems” was the least affected^17^^,^^26^^,^^39^^,^^41^^,^^42^. In these regards, and agreeing with Silva et al.^20^, the difference in domains may be attributed to the cultural backgrounds of the studied populations, namely Latinos and Americans, as the first tend to be more outgoing than the latter.

In this study, 74.6% of the parents gave the maximum score (7 - always) when asked if they were worried with the overall health of their children, suggesting the the quality of their children's sleep is a topic of great concern for them.

The baseline mean score of the OSA-18 questionnaire in this study was a little lower (77.9) than what Silva et al.^20^ reported (82.8), rated as major impact. This probably occurred as a result of the shorter duration of complaints seen in our study. No difference was observed between genders, either for total scores of the scores in each OSA-18 domain, as also reported by other authors^20^^,^^26^^,^^39^^,^^40^.

One study found major impact on QOL in 37% of the children with OSAS and moderate impact in 35%, and no statistically significant differences between OSAS and PS groups^38^. This study found similar results, with major impact on QOL seen in 53.3% of the children with OSAS, moderate in 40%, and no statistically significant differences between OSAS and PS groups (*p* = 0.18).

Mitchell & Kelly^38^ showed that the OSA-18 total baseline score was higher in the OSAS group (72.8), but with no statistically significant difference in relation to the PS group (69.4). In our study higher mean scores were seen in the OSAS group (83) when compared to the PS group (76.2), albeit without statistical significance (*p* = 0.08). But statistically significant difference was observed when OSAS and POS groups were compared for OSA-18 domain “physical suffering” (*p* = 0.04).

Many authors have described physical disturbances, behavioral disorders, and cases of attention deficit in children with sleep obstructive respiratory symptoms caused by adenotonsillar hypertrophy, who experienced short and long term QOL improvements after adenotonsillectomy^9^^,^^18^^,^^39^^,^^43^.

Moderate and major QOL involvement was seen in over 89% of the children enrolled in this study. Social and demographic variables such as gender, age, time with symptoms, household income, age of caregivers, caregiver level of schooling, and time spent by caregivers at school were not statistically different when OSAS and PS groups were compared, as also reported by Mitchell & Kelly^38^.

This study presented a limitation, the lack of a control group with healthy children. This fault, however, does not invalidate the study, as its main goal was achieved, i.e., assess the quality of life of children affected by a specific disease group (sleep-disordered breathing) that encompasses OSAS and PS. It is true that respiratory events caught on PSG may vary from night to night, but it is worth remembering that significant SDB indices are smaller in the pediatric population, and thus a large number of events is not required for OSAS to be characterized.

It was with this purpose that a specific survey to assess impact on quality of life - the OSA-18 questionnaire - was answered by the children's caregivers to compare OSAS (apnea) and PS (no apnea) groups. Another limitation was that many patients reported repetition tonsillitis and nasal symptoms such as rhinorrhea and frequent sneezing, which could be associated with allergic rhinitis and also worsen their sleep during episodes and affect quality of life. Future controlled studies with children without sleep disorders and with chronic tonsillitis and allergic rhinitis should be considered.

The subjectivity resulting from the use of questionnaires in this study was minimized through one objective measure, full-night PSG. PSG sessions were carried out in this study despite the difficulty our children had having access to the test and the complexity of PSG testing in itself. In this study, all children were mouth breathers with low nasal airflow. Therefore, a nasal thermistor was used during PSG similarly to what Izu et al.^35^ described.

Many Brazilian papers have looked into the impacts on the quality of life of children selected for adenoidectomy or adenotonsillectomy, and all found QOL involvement at first and improvement after surgery^20^, ^21^, ^22^, ^23^, ^24^, ^25^.

The effects of OSAS in the overall quality of life of children requires more attention from public health policy makers^18^.

Untreated pediatric SDB may lead to adverse present and future consequences to the affected individuals, their families, and society. Unfortunately, many individuals still remain undiagnosed and untreated.

This study enrolled underprivileged children with SDB and found compromised quality of life among subjects with OSAS and primary snorers.

## CONCLUSION

The quality of life of children with sleep-disordered breathing is compromised. The most affected domains on questionnaire OSA-18 were “concern of persons in charge”, “sleep disturbance”, and “physical suffering”, the last item being rated more highly by subjects with apnea than primary snorers.
